# Terpene-Enriched CBD oil for treating autism-derived symptoms unresponsive to pure CBD: Case report

**DOI:** 10.3389/fphar.2022.979403

**Published:** 2022-10-28

**Authors:** Noa Raz, Iso Heller, Titti Lombardi, Giorgio Marino, Elyad M. Davidson, Aharon M. Eyal

**Affiliations:** ^1^ Bazelet Medical Cannabis Group, Or Akiva, Israel; ^2^ THC Lab Srl, Mirandola, Italy; ^3^ Neurology and Psychiatry Private Clinic, SIPI, Naples–Campania, Italy; ^4^ Department of Anesthesiology, CCM and Pain Relief, Hadassah Hebrew University Hospital, Jerusalem, Israel

**Keywords:** autism, cbd, terpenes, terpene-enriched, treatment efficacy, case report, agitation

## Abstract

Cannabidiol (CBD) rich products are successfully used in some countries for treating symptoms associated with autism spectrum disorder (ASD). Yet, CBD provides insufficient intervention in some individuals, or for some characterizing symptoms of ASD, raising the need for improved compositions. The current study presents a case wherein pure CBD was sufficient for treating ASD during childhood and early adolescence. However, it became insufficient during puberty accompanied by increased hyperactivity, agitation, and frequent severe aggressive behavior. Increasing the CBD dose did not result in significant improvement. Enriching the pure CBD with a carefully selected blend of anxiolytic and calming terpenes, resulted in gradual elimination of those aggressive events. Importantly, this was achieved with a significantly reduced CBD dose, being less than one-half the amount used when treating with pure CBD. This case demonstrates a strong improvement in efficacy due to terpene enrichment, where pure CBD was not sufficient. Combined with terpenes’ high safety index and the ease with which they can be incorporated into cannabinoid-containing products, terpene-enriched CBD products may provide a preferred approach for treating ASD and related conditions. The careful selection of terpenes to be added enables maximizing the efficacy and tailoring the composition to particular and changing needs of ASD subjects, e.g., at different times of the day (daytime vs nighttime products).

## Background

Autism spectrum disorder (ASD) is a group of heterogeneous neurodevelopmental disorders, commonly characterized by an early-onset impairment in social interactions and communications, accompanied by restricted or repetitive patterns of behavior. Approximately 50% of ASD children and adolescents demonstrate behavioral difficulties, including aggression, self-injury, tantrums and hyperactivity. Anxiety disorders are highly prevalent, ranging between 42–79% of the ASD population (Kent and Simonoff, 2017). Sleep disorders, gastrointestinal disorders and immune dysfunction are also quite frequent. The incidence of ASD is about 1–2.5% (Aran and Cayam-Rand, 2020; Su et al., 2021).

Currently, no established pharmacological treatment for the core symptoms of ASD is available. Some medications were approved by the United States Food and Drug Administration (FDA) to treat associated symptoms, including irritability, aggression and behavioral disorders. Commonly used medications include psychotropic drugs (such as atypical antipsychotics), selective serotonin reuptake inhibitors (SSRI’s), stimulants or anxiolytics. However, long-term use of these drugs may cause serious side effects, including sedation, apathy and weight gain. Additionally, these medications are frequently insufficient to provide a significant improvement. The most common treatment for ASD nowadays comprises behavioral and educational therapies; however, these are inadequate or unsuitable for many ASD individuals (Canitano and Scandurra, 2008; Wink et al., 2010; Quintana et al., 2021).

An increasing number of studies has pointed to the potential role of cannabidiol (CBD), the second most abundant cannabinoid in the cannabis plant, in treatment of multiple symptoms associated with ASD (Gu, 2017; Aran et al., 2019a; Bar-Lev Schleider et al., 2019; Barchel et al., 2019; Su et al., 2021). Accordingly, CBD-based products are increasingly prescribed for relieving ASD-associated symptoms. Yet, a considerable variation between countries exists. Epidolex, a pure CBD preparation was recently approved by FDA for treating seizures associated with Lennox-Gastaut syndrome, Dravet syndrome or tuberous sclerosis complex. Given their comorbidity with ASD, CBD is currently used for treating a sub-population of the ASD patients.

### The endocannabinoid system in ASD

The endocannabinoid system (ECS), the endogenous target of cannabis active pharmaceutical ingredients (APIs), plays an important role in central nervous system (CNS) development, in synaptic plasticity, and in the response to endogenous and environmental insults. The ECS has a major role in controlling and regulating multiple physiological systems. Particularly, the ECS has been shown to participate in the regulation of social reward behavior, plausibly by oxytocin-dependent activation of the ECS (Wei et al., 2015), and was suggested, by both animal models and clinical studies, to be a potential target for the treatment of autism. (Aran et al., 2019b; Su et al., 2021; Zou et al., 2021).

The ECS comprises cannabinoid receptors, endocannabinoids as ligands, and enzymes responsible for the synthesis and degradation of these endocannabinoids. 2-Arachidonoyl glycerol (2-AG) and arachidonoyl ethanolamine (Anandamide, AEA) are the best-studied endocannabinoids. They have different synthesis and degradation conducted by different enzymatic pathways and impart distinct physiological and pathophysiological roles. The ECS involves multiple cannabinoid receptors, the most common being cannabinoid receptor type 1 (CB1) and cannabinoid receptor type 2 (CB2), which are mostly expressed in the central nervous system and throughout the immune system, respectively. Other receptors include the transient receptor potential (TRP) channels, and the peroxisome proliferator activated receptors (PPAR’s). In addition to their activation by endogenous cannabinoids (endocannabinoids), the ECS receptors are responsive to exogenous cannabinoids, which could be of two types, phytocannabinoids - extracted from the cannabis plant, and synthetic cannabinoids (e.g. (Pacher et al., 2006; De Petrocellis and Di Marzo, 2009; Lu and MacKie, 2016)).

Studies have pointed to a possible deficiency of the ECS in ASD, which is plausibly involved in known ASD comorbidities, including anxiety, cognitive impairments and sleep disturbances. Alterations of the ECS have been demonstrated in several animal models of ASD. In some of these models, activating the ECS reversed the social deficits. Recent studies have demonstrated lower serum levels of AEA (Zamberletti et al., 2017; Karhson et al., 2018; Aran et al., 2019b), as well as of PEA (N-palmitoylethanolamine) and OEA (N-oleoylethanolamine); two endocannabinoid-like compounds in children with ASD as compared to controls. These may suggest that impaired signaling of AEA (and possibly of their associated compounds) is involved in the pathophysiology of ASD. However, further studies are required to support such claims (Aran et al., 2019b).

### Cannabis active pharmaceutical compounds

Cannabis is a multifaceted plant with hundreds of different chemical entities, including cannabinoids, terpenes and flavonoids. Among the cannabinoids, the most abundant in terms of content and studies, are delta-9-tetrahydrocannabinol (THC) and cannabidiol (CBD). THC, the psychoactive cannabinoid, binds with high affinity to the two ECS main receptors, CB1 and CB2. Common effects of THC include euphoria, altered sensory perception and relaxation. Given its psychoactive potential, THC is rarely used in young ASD patients. CBD has a much wider role, potency and receptor binding properties in a variety of conditions. CBD effects include anticonvulsant, anxiolytic, antipsychotic, anti-inflammatory, anti-oxidant and neuroprotective. CBD interacts with many signaling systems, including TRPV1 (Transient Receptor Potential cation channel subfamily V member 1), TRPV2 (Transient Receptor Potential cation channel subfamily V member 2), glycine α3 and α1, adenosine, 5-HT1a (serotonin 1A receptor), GPR55 (G protein-coupled receptor 55) and PPARγ (Peroxisome proliferator- activated receptor gamma) (Pacher et al., 2006; De Petrocellis and Di Marzo, 2009; Gu, 2017). Specifically for the case of ASD, CBD may play a role in the regulation of social cognition and behavior *via* interaction with oxytocin and vasopression signaling at TRPV1 and TRPV2 (Wainwright et al., 2004; Wei et al., 2015). CBD further acts as an antidepressant and an anxiolytic agent, *via* 5-HT1a modulation (Linge et al., 2016), and most importantly, elevates AEA levels, as a modulator of its degraded enzyme, FAAH (Fatty acid amide hydrolase). A recent study (Mastinu et al., 2022) had demonstrated that treating mice with CBD-rich cannabis extract results in increased social and reduced nonsocial behaviors. CBD has a very high toxicity threshold in humans and other species, and no teratogenic or mutagenic effects have been found to be induced by CBD (Larsen, 2020).

CBD is being increasingly used to treat ASD related symptoms (Gu, 2017). Several case-series were reported on treating children having ASD and severe behavioral problems, showing high tolerability and high efficacy of CBD-rich cannabis extracts (e.g. (Aran et al., 2019a; Bar-Lev Schleider et al., 2019; Barchel et al., 2019; Fleury-Teixeira et al., 2019; Hacohen et al., 2022)). CBD-rich extracts were reported to substantially decrease the irritability, rage attacks and anxiety in most of the participants and to improve the social deficits in about one-half of the subjects. Improvement in sleep, and increased concentration were also documented. These results were recently supported by a blinded placebo-controlled clinical trial on ASD children (Aran et al., 2021).

Terpenes belong to a class of aromatic compounds common in flowers, fruits and vegetables. Apart from their aromatic features, multiple pre-clinical and clinical trials have pointed to the pharmacological effects of specific terpenes on multiple physiological and psychological conditions. Some terpenes act on multiple receptors and neurotransmitters, e.g., as serotonin uptake inhibitors (as does Prozac), norepinephrine and dopamine activity modulators (as do tricyclic antidepressants, monoamine oxidase inhibitors and bupropion), and GABA (Gamma-aminobutyric acid) receptors modulators (as do baclofen and the benzodiazepines) (Russo, 2011; Nuutinen, 2018). Selected terpenes have also been shown to activate receptors of the ECS, including CB2 (Esraa et al., 2020), TRPs (Meotti et al., 2014) and PPARs (Goto et al., 2010; Galaj et al., 2021). Recently, the role of specific terpenes as agonists of the main ECS receptor, CB1, was also demonstrated (LaVigne et al., 2021). The role of selected terpenes as modulators of the activity of cannabinoids at the ECS receptors, sometimes referred to as the “entourage effect” is widely discussed and debated nowadays (Russo, 2011; Lewis et al., 2018; Santiago et al., 2019; Finaly et al., 2020; LaVigne et al., 2021).

In this study we tested whether CBD oils enriched with selected terpenes provide improved therapeutic effects in an ASD agitated adolescent, where pure CBD treatment was found to be insufficient. Two terpene-enriched CBD oils were administrated, the first–suitable for daily functioning, and the second, having higher calming value, for night use or for time intervals wherein the ASD adolescent tends to be more agitated. The study compares the efficacy of pure synthetic CBD oil to the efficacy of the same synthetic CBD oil enriched with selected terpenes, providing a direct, first-ever examination of terpenes-CBD combination for treating ASD related functions.

## Case study

G is an Italian 17 years old teenager. G had normal development up to the age of three, at which age he stopped talking and started presenting an increased hyperactive and stereotypical behaviors, including hand flapping and up and down movements. In June 2008, G was diagnosed with regressive autism. Diagnosis took place at the University Policlinic Hospital in Naples. During childhood and early adolescent years, G’s main behavioral and cognitive deficits included low socialization at school and major concentration difficulties, leading to the inability to acquire reading and writing skills, and communication restricted to basic daily needs (requesting drink, food and sleep). G was treated by behavior and cognitive therapies, including psychomotricity at earlier ages, and ABA- Apply Behavior Analysis. Speech therapy was also employed to improve verbal and social capabilities. At the age of eight, G’s hyperactivity had notably increased, leading his physician to prescribe medicinal treatment with Neuleptil (also known as Perithiazine; indicated for the treatment of psychotic disorders, severe anxiety and stress, impulsivity and aggression) at a daily dose of 4.5 mg (9 drops), which was increased to 15 mg (30 drops) at the age of 13 years.

On February 2018, at the age of 13, G’s first major aggressive event occurred, as described by his father: “He woke up with red eyes, started to scream and punched his head. Whenever anyone tried to stop him, G started hitting them”. While looking for a therapeutic alternative, G’s parents were introduced to CBD and its potential role in reducing aggressive behavior in ASD. On August 2018, after contacting a medical cannabis prescribing physician and consulting a specialized laboratory (THC Lab), G was first prescribed a CBD-rich cannabis extract containing 2.5% CBD. This was later exchanged with pure synthetic CBD oil at the same concentration. CBD oil was first administrated at a daily dose of 15 drops (12 mg CBD), corresponding to 0.34 mg CBD/kg bw (body weight) per day. CBD accompanied the Neuleptil treatment. This was found beneficial, completely eliminating aggression and leaving G happy and calm.

CBD treatment was efficient for 3 years, until G turned 16 years old. From April 2021, with puberty, G became highly agitated. In order to control his symptoms, the dose of pure CBD was raised, reaching 27 drops (21.6 mg CBD), corresponding to 0.48 mg CBD/kg bw per day. However, the increased dose did not result in improved efficacy. Aggression had increased, reaching more than two major aggressive events per week. Aggressive events were severe (scored eight to nine on a 0–10 subjective aggressive scale, by G’s parents). As described by G’s father: “G used to wake up with his eyes red, starting to scream and punching his head. As we avoided calling the hospital for Valium treatment, G started to destroy his bedroom, bed, clothes, lamps and souvenirs, as well as to hit nearby doors and windows. During rage attack he was even able to pull out the toilet bowl from the floor”.

At this time, and in order to try controlling his aggressive attacks, Neuleptil was replaced by Abilify at a daily dose of 7.5 mg. Abilify, also known as aripiprazole, belongs to the group of atypical antipsychotics, acting on dopamine and serotonin receptors in the brain. Abilify is used for the treatment of restlessness associated with ASD in children aged 6–17 years. At times when aggression had peaked, and to control or terminate the attacks, Tavor (Lorazepam, a benzodiazepine medication, used to treat anxiety disorders, disrupted sleep and severe agitation) was additionally administrated. Administration of Tavor was efficient in terminating the attacks, but further resulted in strong side effects, turning G highly depressed and sad “with grim face” for two consecutive days.

At this time, G’s parents had intensively looked for an alternative, which would reduce aggression in their child, while keeping him happy and functioning. They had consulted with THC Lab and were introduced to terpene-enriched cannabis products, manufactured in Israel by Bazelet medical cannabis company. G’s regular CBD oil (pure synthetic CBD at a concentration of 2.5%) was enriched with one of two proprietary terpene blends, composed of terpenes demonstrated in pre-clinical and clinical trials to produce anxiolytic and calming effects. These included alpha pinene, limonene, linalool, beta caryophyllene and nerolidol. One of these two terpene blends was provided for daily use, and the other - for night use, or for times when G was highly agitated. Overall terpene concentration was 0.15% of the oil. This terpene concentration was chosen to reach 0.15/2.5 terpenes to cannabinoids weight/weight ratio, being in the lower range of terpene content in most cannabis plants (Lewis et al., 2018).

**FIGURE 1:**
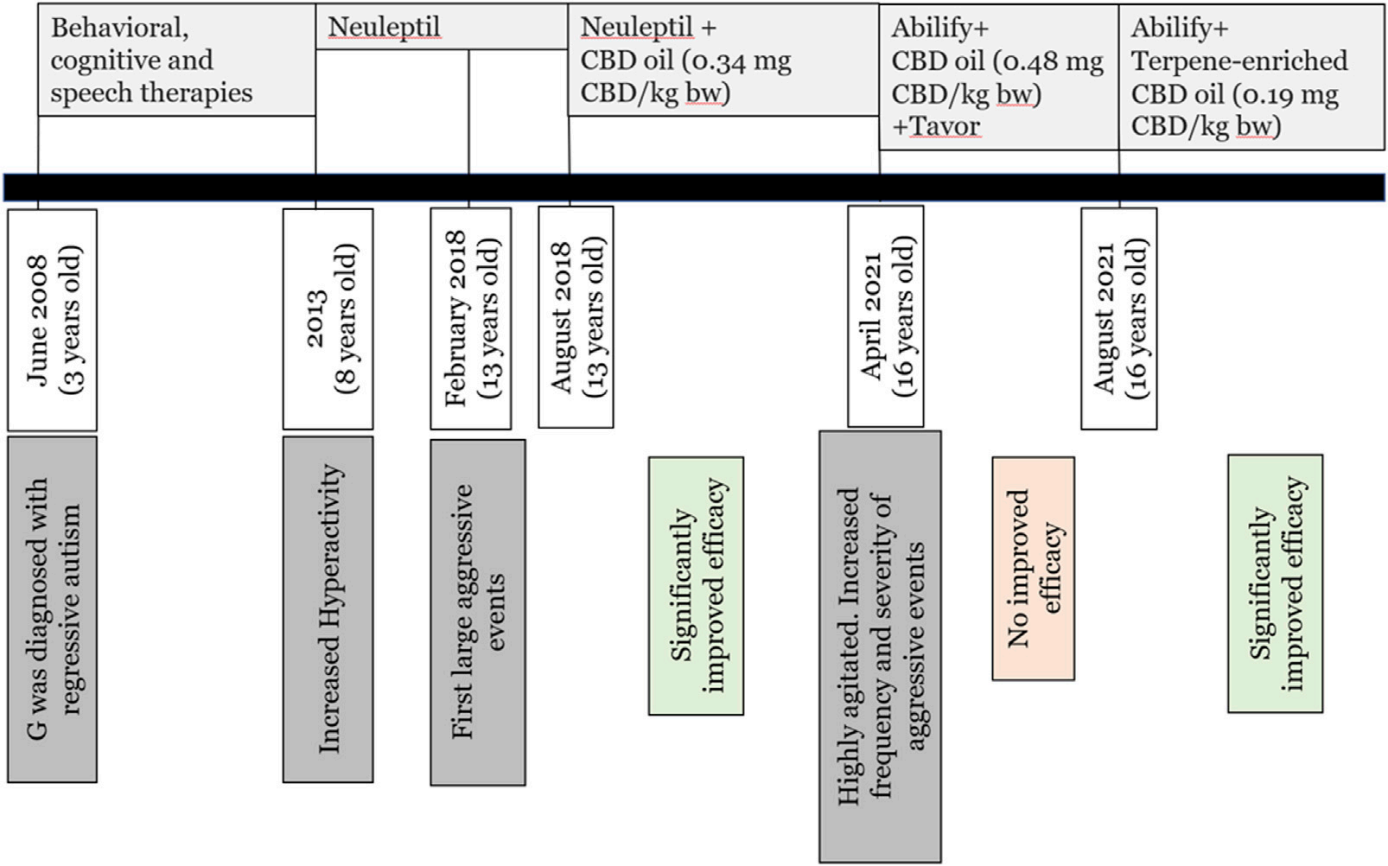
Timeline. White bars represent important timepoints along G’s development. Dark gray bars represent triggering event. Light gray horizonal bars represent given treatment. Light green/orange bars represent efficacy. Time scale is not linear.

In mid-August 2021, G was firstly treated with the terpene -enriched CBD oils. The starting dose was 14 drops daily (given at three timepoints), corresponding to 11.2 mg CBD a day (0.19 mg/kg bw). This dose was found to be sufficient throughout the treatment period, and no increase in dose was required, indicating that the terpene-enriched CBD oil was more potent (Terpene-enriched CBD treatment was accompanied the 7.5 mg daily dose of Abilify, as before) (See Figure 1 for timeline summary).

**FIGURE 2:**
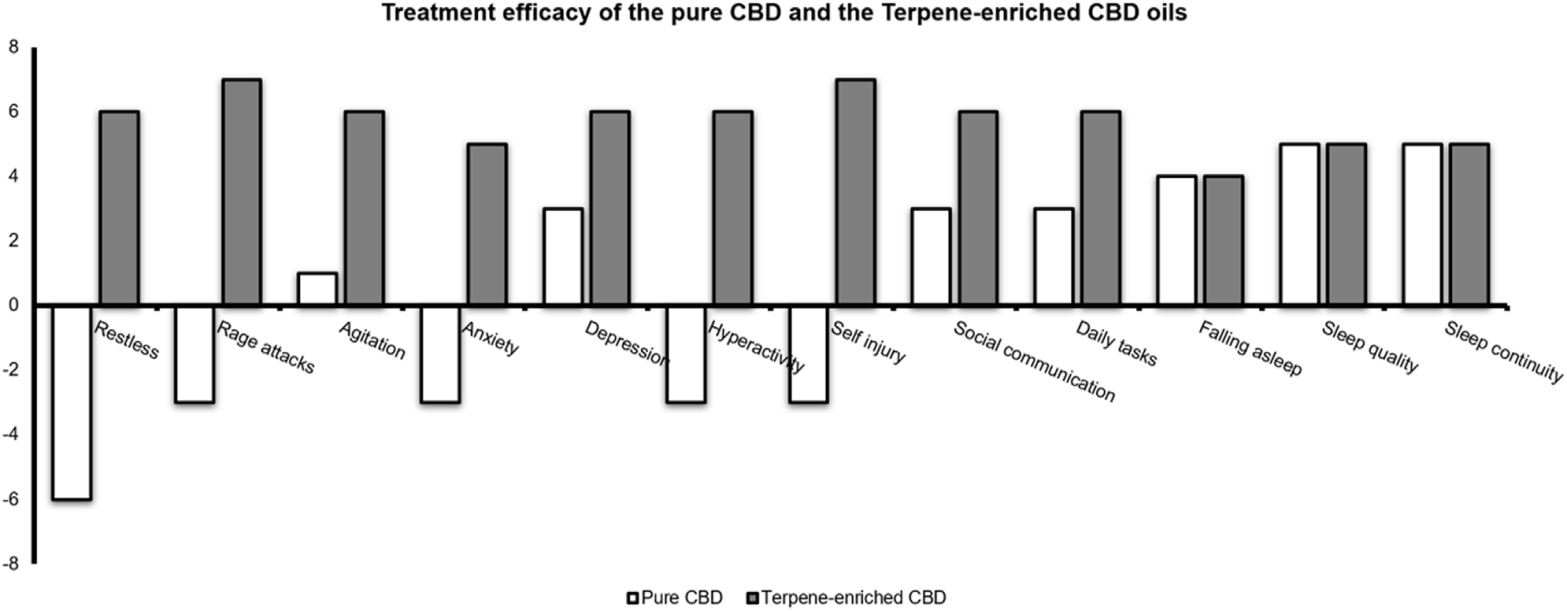
Treatment efficacy of the pure CBD (white bars) and the Terpene-enriched CBD (gray bars) oils. G’s parents were asked to rate symptoms’ severity in their child following CBD oil treatment (“How would you evaluate your child’s symptom severity following oil treatment (how did the oil affected your child in regard the following symptoms)?“. A self-reported questionnaire, see Supplementary data). This was evaluated separately for the pure CBD and the terpene-enriched CBD oils.

Since August 2021, G has completed 9 months of treatment with terpene-enriched CBD oil. As can be seen in Table 1 and in Figure 2, aggression was significantly reduced, from two major aggressive events per week during treatment with synthetic CBD oil, to a complete elimination of aggressive events during treatment with the same synthetic CBD oil enriched with the selected terpene blends. Reduction of aggression was gradual, as seen in Table 1. Importantly, efficacy was achieved at a CBD dose of less than a half the amount of CBD in pure CBD oil (0.19 mg/bw vs 0.48 mg/bw, respectively). Given the significant reduction in aggression, treatment with Tavor was reduced dramatically. In addition to the notable decrease in aggression, G’s parents report improved verbal capabilities, presented as increase in fluent and spontaneous speech, as well as increased levels of humor.

**TABLE 1:** Frequency of large aggressive events and associated Tavor treatment.

Age	Treatment	Frequency of large aggressive events per month (average)		Frequency of administrating Tavor per month (average)
8–13 years old	Neuleptil (4.5 mg daily)	None		None
13–16 years old	Neuleptil (up to 15 mg daily)	4		None
	Neuleptil (up to 15 mg daily) + 2.5% CBD oil (0.34 mg CBD/kg bw daily)	None		None
16–17 years old	Abilify (7.5 mg daily) + 2.5% CBD oil (0.48 mg CBD/kg bw daily)	8		8
	Abilify (7.5 mg daily) + 2.5% Terpene- enriched CBD oil (0.19 mg CBD/kg bw daily)	1–2nd months (September–October 2021)	4	4
		3rd month (November 2021)	3	3
		4th month (December 2021)	2	2
		5–6th months (January–February 2022)	0.5	0.5
		7–9th months (March–April 2022)	None	None

## Discussion

This case demonstrates the benefit of terpene-enriched CBD oil for treating aggression associated with ASD in an agitated adolescent. Enrichment of CBD with selected terpenes increased CBD potency, providing a therapeutic response wherein CBD alone had insufficient effect.

CBD oil (2.5%), in combination with Neuleptil, provided a reasonable solution for G during his early teen years (13–16 years old). During this period, the average daily CBD dose was about 0.34 mg CBD/kg bw. However, following puberty, new severe and frequent aggressive behaviors occurred, and the medicinal care became insufficient. Raising the CBD dose to 0.48 mg CBD/kg bw did not result in improved efficacy. However, enrichment of the CBD oil with 0.15% of selected terpenes successfully reduced severe aggressive attacks. Importantly, this was achieved with no side effects (in contrast to treatment with the regimen Tavor), leaving G happy and functioning. The desired therapeutic response was obtained at reduced CBD dosages (0.19 mg/kg bw).

A possible support for terpenes facilitation of CBD is derived from studies comparing the efficacy of CBD rich extracts to that of pure CBD at the same doses. Improved efficacy of the former was demonstrated in reducing inflammation and pain in mice (Gallily et al., 2015), and in alleviating seizures in patients with refractory epilepsy (Pamplona et al., 2018), wherein, CBD rich extracts were more effective at lower doses, and resulted in fewer side effects. The term “entourage effect” was coined for such improved effect of the multi-component extract.

Importantly, however, since extracts naturally contain multiple compounds (beyond terpenes, such as other cannabinoids and flavonoids), their superiority over pure CBD, cannot directly confirm the role of terpenes in improving potency. The current study directly compared the effect of synthetic CBD to that of the same synthetic CBD with the only addition of selected terpene blends. No minor cannabinoids or other compounds were included. As such, the current study provides a strong, first-ever, support for the role of selected terpenes in improving CBD therapeutic effect.

The increased efficacy of the selected terpene-enriched CBD oil in our results may relate to the terpenes’ own therapeutic effects. Indeed, several publications have pointed to the role of selected terpenes in treating anxiety, in reducing aggressive behavior, and in improving social interactions (e.g. (Linck et al., 2010; Russo, 2011; De Sousa et al., 2015; Lewis et al., 2018; Nuutinen, 2018; Ferber et al., 2019)). Other studies have demonstrated the role of terpenes in augmenting cannabis anxiolytic profile (Kamal et al., 2018; Mastinu et al., 2022). However, given the low, plausibly sub-therapeutic concentrations of the terpenes in these studies, an alternative mechanism is suggested here. Accordingly, the enhanced pharmacological effect of the terpene-enriched CBD oil results from a modulatory effect of those selected terpenes on the interaction of CBD with the ECS receptors.

Despite some terpene content in cannabis extracts, it is important to note that a major part of cannabis terpenes is lost during standard cannabis preparations. In fact, the majority of the most abundant cannabis terpenes (e.g., alpha and beta pinene, myrcene, limonene and linalool) are nearly completely absent in many cannabis extracts due to evaporation during the extraction and the decarboxylation process (Ternelli et al., 2020; Eyal et al., 2002; Raz et al., 2022). This terpene loss questions the common use of “whole plant” or “full spectrum” terms when applied to cannabis extracts. Due to this loss, treatment with standard (generic) cannabis extracts (sometimes referred to as “chemovaric extracts”) may provide a suboptimal treatment.

Alternatively to selecting for the right chemovar/extract, selected terpenes may be easily added into cannabinoid products (pure or extracts) *via* a designated industrial procedure. Terpenes are defined as GRAS (generally recognized as safe) by the Food and Drug Administration (FDA) and by the Flavor and Extract Manufacturers Association (FEMA) organizations (Femaflavor,, United States Food and Drug Administration). And may thus be safely used in daily consumed products.

While the practical addition of terpenes is easily performed in industrial process, the selection of terpenes to be added is far from being obvious. Different terpenes have various therapeutic effects. For example, while some have sedative/calming effects, others have stimulatory effects. These differences are useful, for instance, to produce terpene-enriched cannabinoid products for day vs night use. Additionally, different terpenes may have different modulatory effects on cannabinoid activity at the ECS. Selection of the most suitable terpenes to be added is based on a thorough research and study of their mechanisms of action, on analysis of patients’ responses, and on *in vitro*, pre-clinical and clinical studies.

To summarize, the low toxicity levels of terpenes, the ease with which they can be incorporated into cannabinoid products in an industrial setting, and above all–the strong therapeutic benefit of some of them in conjunction with CBD, as demonstrated herein, highlights the great therapeutic implication of terpene-enriched CBD products in treating ASD and associated conditions. Addition of the selected terpenes reduced the required CBD dosages by more than a half and critically, resulted in a major reduction in aggressive behavior without notable side effects.

Obviously, a placebo controlled double blind clinical trials are needed to further evaluate the superior effect of terpene-enriched CBD oil in ASD. For now, we were privileged to contribute to at least one family dealing with ASD. As summarized by G’s father: “by using CBD enriched with these terpenes, I am able to keep G with us in one house without institutionalizing him. For me, that is the real blessing. Terpene-enriched CBD treatment relieves us from reaching neuroleptic drugs treatment, turning so many ASD adolescents daze and confused. I hope that by this case we can show the way for all ASD families to not leave CBD treatment if after some time it turns out insufficient, but rather to modulate it *via* enrichment with appropriate terpenes”. Terpene-enriched products are already manufactured and marketed in the Israeli market, e.g. (Raz and Eyal, 2018; Eyal and Raz, 2017; Raz et al., 2020).

## Data Availability

The original contributions presented in the study are included in the article/supplementary material, further inquiries can be directed to the corresponding author.
