# Supplementary material for: Terpene-Enriched CBD oil for treating autism-derived symptoms unresponsive to pure CBD: Case report
Source: Front Pharmacol. 2022 Oct 28;13:979403. doi: 10.3389/fphar.2022.979403 (PMC9649447; doi:10.3389/fphar.2022.979403)
Supplement: Supplementary file 1 [file DataSheet1.PDF]

Autism Questionnaire

Date _______________

Oil given: ________________.

1. **How would you evaluate your child's symptoms severity following oil treatment (how did the oil affected your child in regard the following symptoms)?**

- **Restlessness**

Not relevant	Exterme deterioration	Severe deterioration			Moderate deterioration			Slight deterioration			No Change	Slight improvement			Moderate improvement			Great improvement			completely gone
-	-10	-9	-8	-7	-6	-5	-4	-3	-2	-1	0	1	2	3	4	5	6	7	8	9	10

- **Rage attacks**

Not relevant	Exterme deterioration	Severe deterioration			Moderate deterioration			Slight deterioration			No Change	Slight improvement			Moderate improvement			Great improvement			completely gone
-	-10	-9	-8	-7	-6	-5	-4	-3	-2	-1	0	1	2	3	4	5	6	7	8	9	10

- **Agitation**

Not relevant	Exterme deterioration	Severe deterioration			Moderate deterioration			Slight deterioration			No Change	Slight improvement			Moderate improvement			Great improvement			completely gone
-	-10	-9	-8	-7	-6	-5	-4	-3	-2	-1	0	1	2	3	4	5	6	7	8	9	10

- **Anxiety**

Not relevant	Exterme deterioration	Severe deterioration	Moderate deterioration	Slight deterioration	No Change	Slight improvement	Moderate improvement	Great improvement	completely gone

-	-10	-9	-8	-7	-6	-5	-4	-3	-2	-1	0	1	2	3	4	5	6	7	8	9	10

- **Depression**

Not relevant	Exterme deterioration	Severe deterioration			Moderate deterioration			Slight deterioration			No Change	Slight improvement			Moderate improvement			Great improvement			completely gone
-	-10	-9	-8	-7	-6	-5	-4	-3	-2	-1	0	1	2	3	4	5	6	7	8	9	10

- **Seizures**

Not relevant	Exterme deterioration	Severe deterioration			Moderate deterioration			Slight deterioration			No Change	Slight improvement			Moderate improvement			Great improvement			completely gone
-	-10	-9	-8	-7	-6	-5	-4	-3	-2	-1	0	1	2	3	4	5	6	7	8	9	10

- **Tics**

Not relevant	Exterme deterioration	Severe deterioration			Moderate deterioration			Slight deterioration			No Change	Slight improvement			Moderate improvement			Great improvement			completely gone
-	-10	-9	-8	-7	-6	-5	-4	-3	-2	-1	0	1	2	3	4	5	6	7	8	9	10

- **Hyperactivity**

Not relevant	Exterme deterioration	Severe deterioration			Moderate deterioration			Slight deterioration			No Change	Slight improvement			Moderate improvement			Great improvement			completely gone
-	-10	-9	-8	-7	-6	-5	-4	-3	-2	-1	0	1	2	3	4	5	6	7	8	9	10

- **Self-injury**

Not relevant	Exterme deterioration	Severe deterioration			Moderate deterioration			Slight deterioration			No Change	Slight improvement			Moderate improvement			Great improvement			completely gone
-	-10	-9	-8	-7	-6	-5	-4	-3	-2	-1	0	1	2	3	4	5	6	7	8	9	10

- **Social communication and reciprocity**

Not relevant	Exterme deterioration	Severe deterioration			Moderate deterioration			Slight deterioration			No Change	Slight improvement			Moderate improvement			Great improvement			completely gone
-	-10	-9	-8	-7	-6	-5	-4	-3	-2	-1	0	1	2	3	4	5	6	7	8	9	10

2. How would you evaluate the ability of your child to manage daily tasks (such as getting dressed, showering, brushing teeth) following oil treatment?

Not relevant	Exterme deterioration	Severe deterioration			Moderate deterioration			Slight deterioration			No Change	Slight improvement			Moderate improvement			Great improvement			completely gone
-	-10	-9	-8	-7	-6	-5	-4	-3	-2	-1	0	1	2	3	4	5	6	7	8	9	10

3. Sleep quality

3.1. How would you evaluate the ability of your child to fall asleep following oil treatment?

Not relevant	Exterme deterioration	Severe deterioration			Moderate deterioration			Slight deterioration			No Change	Slight improvement			Moderate improvement			Great improvement			Perfect
-	-10	-9	-8	-7	-6	-5	-4	-3	-2	-1	0	1	2	3	4	5	6	7	8	9	10

3.2. How would you evaluate the sleep quality of your child following oil treatment?

Not relevant	Exterme deterioration	Severe deterioration	Moderate deterioration	Slight deterioration	No Change	Slight improvement	Moderate improvement	Great improvement	Perfect

-	-10	-9	-8	-7	-6	-5	-4	-3	-2	-1	0	1	2	3	4	5	6	7	8	9	10

3.3. How would you evaluate the sleep continuity of your child following oil treatment?

Not relevant	Exterme deterioration	Severe deterioration			Moderate deterioration			Slight deterioration			No Change	Slight improvement			Moderate improvement			Great improvement			Perfect
-	-10	-9	-8	-7	-6	-5	-4	-3	-2	-1	0	1	2	3	4	5	6	7	8	9	10

4. **Had your child experienced any adverse effects following CBD oil usage?**

No

Yes, slight adverse effects

Yes, severe adverse effects

Please elaborate ____.
